# Supporting dynamic change detection: using the right tool for the task

**DOI:** 10.1186/s41235-016-0033-4

**Published:** 2016-12-19

**Authors:** Benoît R. Vallières, Helen M. Hodgetts, François Vachon, Sébastien Tremblay

**Affiliations:** École de Psychologie, Université Laval, Pavillon Félix-Antoine-Savard, 2325, rue des Bibliothèques, Québec, QC G1V 0A6 Canada

**Keywords:** Change blindness, Dynamic decision-making, Change History EXplicit (CHEX), Eye tracking, Decision support system

## Abstract

Detecting task-relevant changes in a visual scene is necessary for successfully monitoring and managing dynamic command and control situations. Change blindness—the failure to notice visual changes—is an important source of human error. Change History EXplicit (CHEX) is a tool developed to aid change detection and maintain situation awareness; and in the current study we test the generality of its ability to facilitate the detection of changes when this subtask is embedded within a broader dynamic decision-making task. A multitasking air-warfare simulation required participants to perform radar-based subtasks, for which change detection was a necessary aspect of the higher-order goal of protecting one’s own ship. In this task, however, CHEX rendered the operator even more vulnerable to attentional failures in change detection and increased perceived workload. Such support was only effective when participants performed a change detection task without concurrent subtasks. Results are interpreted in terms of the NSEEV model of attention behavior (Steelman, McCarley, & Wickens, Hum. Factors 53:142–153, 2011; J. Exp. Psychol. Appl. 19:403–419, 2013), and suggest that decision aids for use in multitasking contexts must be designed to fit within the available workload capacity of the user so that they may truly augment cognition.

## Significance

Detecting changes in our visual environment is essential to several everyday activities (e.g. driving a car), as well as in safety-critical work settings such as air traffic control and security surveillance. Change blindness—the incapacity to notice visual changes—can negatively impact decision-making and lead to important consequences such as human errors, critical incidents, and, in worse cases, loss of human lives. This current study intends to broaden our understanding of the sources of change blindness and finds that while some changes may go unnoticed because they are never looked at, participants within a complex environment may also be “blind” to changes/events upon which they are fixating. In a multitasking context, attention is stretched and necessarily divided among several subtasks, leaving fewer attentional resources to ensure that a change—although fixated—actually reaches conscious awareness. While change detection support tools are available, their efficacy is dependent upon the available attentional capacity of the user. We demonstrate that one tool (a change history table) designed to improve detection performance within an air traffic control simulated environment, actually worsens detection performance—perhaps due to an increase in workload and subsequently stretched attentional resources that render the changes less likely to be consciously detected. This indicates the importance of testing potential support tools within a dynamic and demanding environment to determine the extent to which they are actually able to improve performance.

## Background

The ability to discern pertinent objects and events in our environment is essential to a multitude of everyday activities, such as driving and air traffic control (St. John, Smallman, & Manes, 2005). A driver, for example, must constantly monitor the unfolding dynamic visual scene for incoming traffic and changing road signs in order to drive safely and avoid hazards (Horswill & McKenna, 2004). The failure to detect task-relevant changes in a visual scene, often referred to as change blindness (CB; Rensink, Regan, & Clark, 1997), is the source of many human errors; in complex and dynamic situations, in particular, the high volume of information to monitor can overload the operator leaving critical incidents unnoticed and in turn reduce decision-making quality (Durlach, 2004; Varakin, Levin, & Fidler, 2004). Graphical user interface add-ons and tools, such as the Change History EXplicit (CHEX; see Smallman & St. John, 2003), have been designed to support change detection, situation awareness and decision-making in such demanding situations. In the current paper, we test whether the efficacy of CHEX, as designed by Smallman and St. John (2003), can generalize to a dynamic and multitasking context, aiming to extend its use beyond the change-detection-only task environment in which it was originally developed. Using a cognitive and holistic approach will enable us to assess the impact of this type of tool on all the aspects of the wider task environment (including change detection performance) and to further investigate the sources of attentional failures in dynamic situations.

The ability to detect a change depends on attentional and memory processes and the efficiency of the visual system to capture visual transients associated with the transition between the pre-change and the post-change states of an object. When the visual system fails to localize such transients, CB is likely to occur (e.g. Simons, Franconeri, & Reimer, 2000). In static visual scenes, techniques such as the flicker, the mud-splash, and the saccade-contingent paradigms have been used to prevent the visual system from capturing these transient signals and thus induce CB (e.g. Henderson & Hollingworth, 2003; O’Regan, Rensink, & Clark, 1999; Rensink et al., 1997; Simons & Ambinder, 2005). CB has also been studied using dynamic visual scenes in more realistic contexts, such as simulated command and control (C2), security surveillance environments, and monitoring geospatial displays (e.g. DiVita, Obermayer, Nugent, & Linville, 2004; Durlach & Chen, 2003; Durlach, Kring, & Bowens, 2008; Stelzer & Wickens, 2006). In complex and dynamic situations, multiple independent objects in the scene are changing frequently, unpredictably, and sometimes simultaneously, making important visual changes difficult to process and detect (Durlach et al., 2008). For example, in one simulated environment, participants were asked to monitor the geospatial display of an airspace and to detect and identify significant changes (e.g. new aircraft appearing on the display). They missed about 13% of all critical changes (St. John, Smallman, Manes, Feher, & Morrison, 2005). In such dynamic visual scenes, transient signals are naturally less detectable than in static scenes given that numerous features in the scene are moving (hence constantly changing) and can act as distractors (Boot, Kramer, Becic, Wiegmann, & Kubose, 2006). Competition between task-irrelevant transients made by distractors and those produced by goal-relevant changes reduce the detectability of critical changes (Vachon, Vallières, Jones, & Tremblay, 2012).

Given that change detection relies on a comparison between the operator’s perception of the current situation and their representation of the past situation, support tools to supplement the operator’s limited memory and attentional capacity should improve the ability to notice these changes. One such tool is CHEX (Smallman & St. John, 2003), a tool developed within the context of an air-warfare task to aid participants in discriminating significant changes from irrelevant changes that occur in an airspace. CHEX automatically detects and logs all changes in an interactive table positioned next to the radar display, thus providing an external aid and a “second chance” to notice an important change that may have previously gone unnoticed. Each row of the table displays the time the change occurred, the identity of the aircraft concerned, and a brief description of the change, while each column can be filtered by the user as needed. Selecting a row highlights that row, circles the location of the related aircraft on the geospatial display, and highlights other changes in the table made by the same aircraft, and vice versa (dynamic visual linking; see St. John & Smallman, 2008). The CHEX permanent repository is an uninterrupted display of situational changes intended to help operators in recovering from situation awareness breakdowns caused by interruptions and multitasking. Situation awareness is the perception of events, the comprehension of their meaning, and the anticipation of their status with respect to time and space (Endsley, 1995). The CHEX display has been applied to complex dynamic situations known to require multitasking and high attentional load (e.g. unmanned vehicle control; St. John & Smallman, 2008; Parasuraman, Cosenzo, & De Visser, 2009; Vachon et al., 2012).

St. John et al. (2005) compared CHEX with four other tools designed principally to attract attention towards significant events in the dynamic visual scene: a static table that chronologically listed the time and nature of each significant change; two types of instant replay; and explicit markers with “pop” sounds to accompany changes. The participants’ only task was to monitor the airspace and to explicitly declare (i.e. detect and identify) all relevant changes as quickly as possible. Results demonstrated that participants using CHEX were faster and more accurate to detect significant changes and made fewer omissions or errors than when using any other tool. The authors concluded that CHEX was superior to other tools as it supported both the detection and the identification components of the participants’ task. Indeed, one utility of this type of CHEX over the other types of tools is that, in addition to signaling or showing what were the changes in the situation, it gives information about what has changed. For example, explicit markers only aided the detection component and participants had to rely on their memory to infer what had happened based upon contextual cues, whereas changes were unobtrusively notified and described in the CHEX table. Indeed, the table format of CHEX appears to provide a good trade-off between information accessibility and distraction by presenting all changes textually in a permanent log, but by not presenting additional change-related information on the already cluttered geo-spatial plot. CHEX thus seems a very useful support tool for detecting changes within a dynamically changing visual scene and could potentially be used to reduce CB in complex C2 environments.

The current paper aims to extend the use of CHEX beyond the change-detection-only task environment in which it was originally developed (see Smallman & St. John, 2003; St. John et al., 2005), to a more ecologically valid context that mimics the multitasking nature of real C2 operations (see Vachon et al., 2012). The objective is to test the general efficacy of the CHEX situation awareness tool to work settings that are the most likely to require such support systems. In this study, the CHEX serves as a change-detection external aid. Although change detection is a central part of many C2 tasks, it is generally only one of several concurrent subtasks. As such, limited cognitive resources needed for noticing these dynamic changes are even more stretched in these circumstances, as various other aspects of the work are also attention demanding (Parasuraman et al., 2009). Another difference between lab studies of CB, as well as the studies of St. John, et al. (2005; Smallman & St. John, 2003), is that C2 operators rarely need to declare changes explicitly on their displays for the purpose of their mission; change detection is assumed when, after a task-relevant change, an appropriate action is taken (Vachon et al., 2012). Little research has been conducted regarding the extent and prevention of CB in multitasking C2 environments whereby change detection is embedded within the higher-order task of the operator (DiVita et al., 2004; Liebhaber & Feher, 2002). In the present study, we use a simulated naval air-warfare task, whereby participants play the role of a radar operator who must detect (and act upon) critical situational changes while also performing a threat-evaluation and weapon-assignment (TEWA) task. Change detection is thus an important subcomponent of the mission’s goals, but it is embedded within a broader dynamic decision-making task. CHEX has proven useful in a number of studies in which change detection was the only task to perform, but for it to be considered a beneficial change detection tool for C2, it must first be validated within the kind of high tempo, multitasking environment that would likely require such a support system.

In our assessment of the impact of decision support on change detection, we seek to discriminate between two different sources of CB: changes missed because they were never attended and changes that were processed to a certain extent but go unnoticed because of a failure of attentional processes (Vachon et al., 2012). More precisely, the pre-attentive source of CB refers to the idea that unattended changes—i.e. those that are never looked at—are less likely to be consciously perceived, hence more likely to remain undetected; i.e. the change was not perceived (e.g. Rensink et al., 1997; Simons & Ambinder, 2005). The attentional source of CB arises when a change is attended at a time when attentional processes are overloaded with many other sources of information to process, leaving insufficient resources for the change to reach consciousness. This failure to detect corresponds to looking at the change without seeing it (e.g. Caplovitz, Fendrich, & Hughes, 2008; Drew, Võ, & Wolfe, 2013; O’Regan, Deubel, Clark, & Rensink, 2000), although in the absence of conscious awareness, this change may be registered implicitly (Beck, Peterson, & Angeline, 2007; Fernandez-Duque & Thornton, 2003). Vachon et al. (2012) provided psychophysiological evidence that fixated changes that remained undetected were nevertheless processed up to a certain level, probably reflecting a (unanswered) call for attention generated by the automatic detection of attended visual transients. Here we broaden the definition of the attentional source of CB to include the memory processes, comprehension (sense-making), and decision-making necessary for change detection. Attention serves as a supervisory control for different cognitive functions (including those involved in change detection; see Baddeley, 1993), by overseeing comparisons between the pre-change and post-change representations stored in memory, the comprehension of the change (awareness of the evolution of the situation), and the decision-making needed to act upon the change (see Lamme, 2003). A change that is not consciously detected may be due to a breakdown at any of these stages, i.e. a lack of attentional resources to either (and among others) enable the encoding of fixated elements, make comparisons in memory, or understand the change that occurred in the situation (Caplovitz et al., 2008).

Considering the distinction between the two sources of CB, we suggest that CHEX has the potential to be a double-edged sword. On the one hand, if participants miss a change because they were attending elsewhere and did not fixate on it (the pre-attentive source of CB), CHEX provides another means to detect that change as information relating to it is displayed in a permanent and dynamically linked table. On the other hand, providing an additional source of visual information to monitor could increase cognitive load detrimentally, particularly if participants’ attentional resources are already close to capacity given the nature of the tasks. As such, this could increase the risk of attentional breakdown and exacerbate the second source of CB.

### The present study

The task used was the Simulated Combat Control System microworld (S-CCS; Lafond et al., 2009; Vachon et al., 2012), a simulation of the essential subset of cognitive activities performed by tactical coordinators aboard Canadian navy frigates. The use of a microworld represents the best compromise between internal and ecological validities; it provides a high degree of experimental control while allowing the re-creation of displays and scenarios that are functionally comparable to those encountered in real C2 operations (Brehmer & Dörner, 1993; Elliot et al., 2004). For Experiment 1, participants were required to perform TEWA processes while also being alert and responding to unexpected critical changes (i.e. an aircraft changing from a non-threatening to a threatening status) which could potentially compromise the safety of the own ship. The TEWA task involved categorizing aircraft appearing on a radar display according to their threat level (threat evaluation) and scheduling retaliatory actions against threatening aircraft (weapon assignment). Participants were required to detect the critical changes as part of the higher-order goal of ensuring that aircraft were categorized appropriately and defending the own ship.

In Experiment 1, change detection was embedded within the TEWA task; participants were not required to explicitly report the changes occurring, but detection of these changes would enable performance of a timely action to protect the own ship. Smallman and St. John’s (2003) CHEX tool was reproduced and integrated within the S-CCS interface and evaluated according to a holistic approach: the efficiency of this decision support system (DSS) included not only its effects on the function it was designed to support—here, change detection—but also on other cognitive functions such as categorization and scheduling (see Lafond, Vachon, Rousseau, & Tremblay, 2010; Vachon, Lafond, Vallieres, Rousseau, & Tremblay, 2011). In this manner, the efficiency of CHEX was determined by its level of CB prevention, as well as by its impact—positive or negative—on threat evaluation performance, defensive effectiveness, and perceived workload. Experiment 2 again used the S-CCS microworld, but this time participants performed an explicit change detection task (akin to that of Smallman & St. John, 2003; see also St. John et al., 2005b), rather than the TEWA task in which change detection is implicitly required. The same version of the CHEX used in Experiment 1 was implemented within the S-CCS interface for Experiment 2. This second experiment served to replicate the conditions in which the CHEX had previously proven effective, but within the same air-warfare simulation used in Experiment 1.

Along with determining the efficacy of CHEX to improve overall change-detection performance, we measured the contribution of the DSS to prevent or reduce the two sources of CB by further subdividing the percentage of undetected changes according to whether they were fixated or not (see Vachon et al., 2012). Non-fixated (i.e. unattended) undetected changes are indicative of the pre-attentive CB—the failure to direct attention towards the change—whereas fixated (i.e. attended) undetected changes would reveal the occurrence of the second source of CB—insufficient amount of attentional resources to consciously detect a change.

**Fig. 1:**
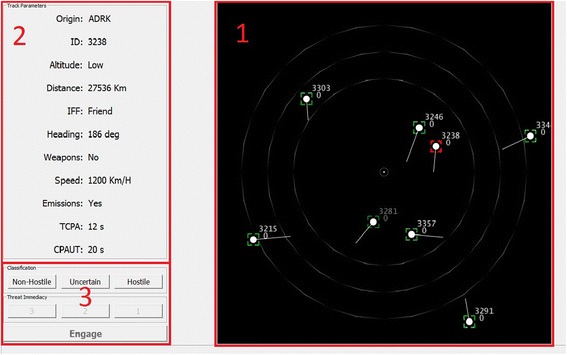
S-CCS interface for the control condition of Experiment 1. Three areas of interest are defined here: (1) aircraft parameter list, (2) radar screen, and (3) action buttons

**Fig. 2:**
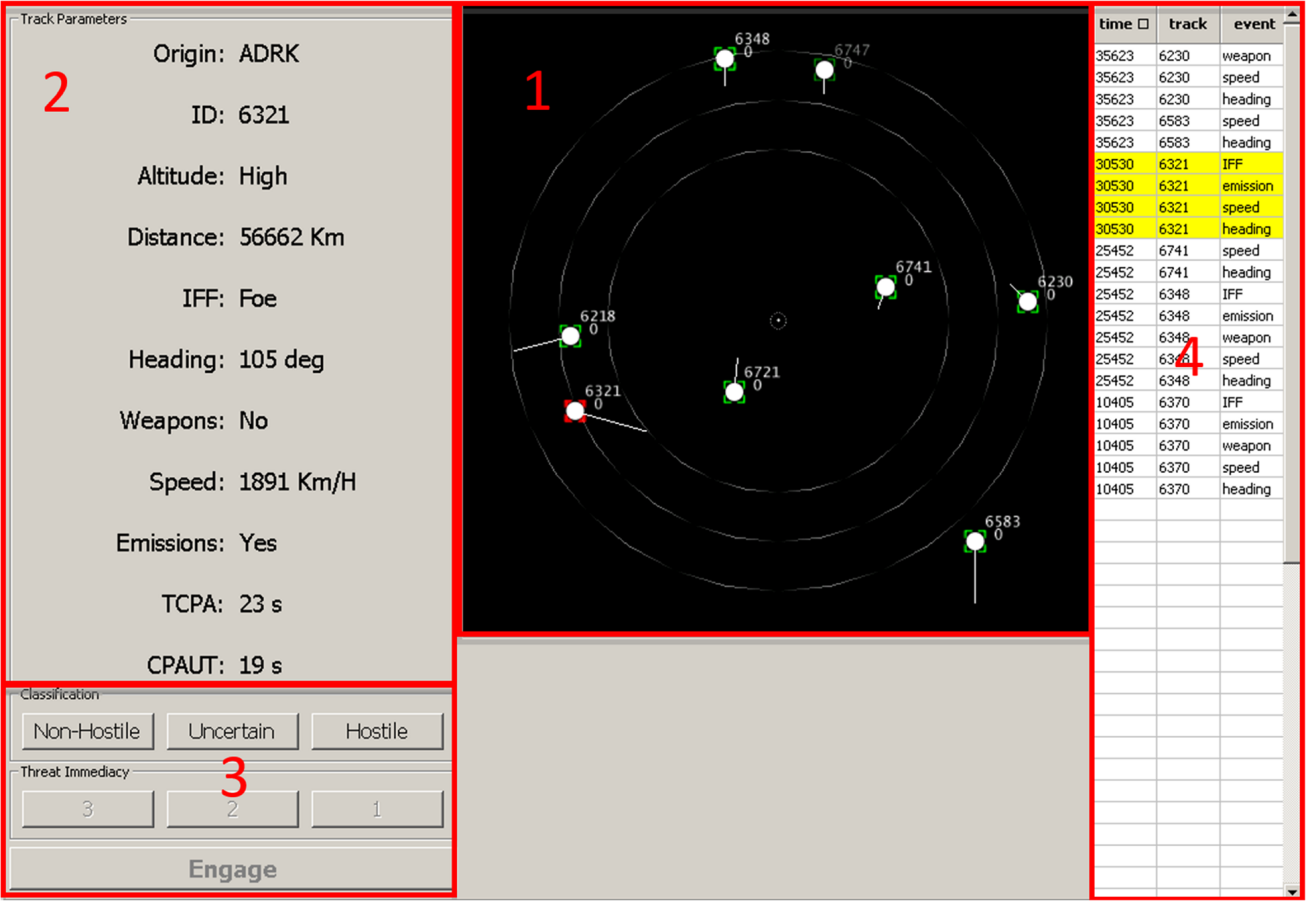
S-CCS interface for the CHEX condition of Experiment 1. CHEX is located to the right of the radar screen

We also examined how CHEX was used by participants through actions on the tool as well as the monitoring and recording of eye movements (e.g. Morrison, Marshall, Kelly, & Moore, 1997; Poole & Ball, 2006). In order to make a usage analysis of the tool, the display was divided into a number of discrete areas of interest (AOIs). Figure 1 illustrates the three AOIs defined in the original S-CCS display, while a fourth AOI was created when CHEX was available on the interface (see Fig. 2). This allowed us to compare the number of aircraft selections made using the tool to those made on the radar. We also extracted metrics related to the attention distribution over the display based on eye movements. Indeed, there is evidence that the overt eye movements to a given location are typically preceded by a displacement of visual selective attention to that location (e.g. McCarley & Kramer, 2008; Rayner, 2009). Hence, the overall dwell time—the sum of all gaze fixation durations within an AOI—as well as the percentage of fixations on the DSS were taken as indicators of attention allocation over the tool (Hauland, 2003). Greater dwell times and percentages of fixation would be indicative of greater devotion of attentional resources to a region of the interface.

Finally, we assessed participants’ subjective level of mental workload according to CHEX and control conditions using the NASA-TLX questionnaire (Hart & Staveland, 1988), a multidimensional rating procedure that produces an overall workload score based on a weighted average of ratings on six subscales. For the purpose of the present study, we focused exclusively on the mental demands and temporal demands subscales, examining the perceived mental load and time pressure in relation to change detection and TEWA performance.

## Experiment 1

Experiment 1 tested the efficacy of the CHEX tool, as it was designed by Smallman and St. John (2003), when the task of change detection was embedded within the broader context of a TEWA task. Based on the method of Vachon et al. (2012), we created a between-subjects experimental design in which we compared the results on change detection, subjective mental workload, and TEWA performance of two groups of participants: a group who performed the task with CHEX and another group with no DSS.

### Method

#### Participants

Fifty-one students at Université Laval (26 men; mean age, 23.04 years), were randomly assigned to either the CHEX (25 participants) or control condition (26 participants). All reported normal or corrected-to-normal vision and received $20 compensation for their participation in a single 2-h experimental session. This study was approved by the Université Laval ethics committee and consent to publish has been obtained from participants.

#### Microworld

The S-CCS simulation (Lafond et al., 2009; Vachon et al., 2012) is dynamic insofar as it evolves according to different scenarios in interaction with the participants’ actions. Scenarios involve multiple aircraft moving in the vicinity of the ship and some of them may represent a potential threat to the frigate. As a tactical coordinator, participants must perform categorization and prioritization of threats, plan and schedule the application of combat power through retaliatory missile firing, and be alert to any changes made by aircraft in the operational space.

The visual display of S-CCS in the control condition can be divided into three sections (see Fig. 1): the radar screen (Area 1), the parameter list (Area 2), and the action buttons (Area 3). The interface is 1024 pixels wide by 768 pixels high which corresponds to the computer monitor resolution. The radar screen visually represents aircraft that move in real time in the ship’s immediate environment with various speeds and directions. Each unselected aircraft is represented as a white dot surrounded by a green square. Participants left-click with the mouse on its icon to select an aircraft. As an aircraft selection visual feedback, the surrounding square turns red to indicate that aircraft is now selected. The parameters list displays information related to the selected aircraft. The action buttons allow participants to assign threat level and immediacy and to neutralize a target aircraft.

#### Task

The participants’ task included four subtasks to be performed concurrently throughout the entire scenario: (1) threat-level assessment, (2) threat-immediacy assessment, (3) neutralization of hostile aircraft, and (4) critical change detection. First, participants were asked to classify all aircraft as they progressively appeared on the radar screen according to three threat levels (non-hostile, uncertain, and hostile). To do so, they had to follow a pre-set classification rule by taking into account five equally weighted parameters displayed in the list: Origin, Altitude, Identification Friend-or-Foe, Military Electronic Emissions, and Detection of Weapons. As each of these parameters could take either a threatening or a non-threatening value, the number of threatening cues established the threat level of an aircraft. An aircraft was (1) non-hostile when 0–1 of his critical parameters were threatening, (2) uncertain when it showed 2–3 threatening cues, or (3) hostile when 4–5 of its critical parameters were threatening. Participants clicked on the corresponding classification button to register their decision, changing the aircraft’s white dot to one of three colors: red for hostile, yellow for uncertain, or green for non-hostile. The threat level of an aircraft could change at any time during a scenario and so participants were instructed to regularly recheck the parameters of already-classified aircraft in case their threat level needed reassessment. The color of the dot representing it only changed in accordance with the classification assigned by the participant and thus provided no cue regarding the occurrence of critical changes.

Second, for all hostile aircraft, participants were instructed to determine the level of threat immediacy based on their temporal proximity from the ship. Participants added the parameter values for the time to closest point of approach and the closest point of approach in units of time to prioritize their actions. Threat immediacy could be high (<15 s from hitting the ship), medium (15–30 s), or low (>30 s). As they made their decision, participants had to click on the corresponding immediacy button (1 to 3, respectively).

Third, because all hostile aircraft were programmed to hit the ship, participants were instructed to defend the ship against hostile tracks by launching an anti-aircraft missile. In real life, of course, there would be many safeguards and intermediate steps before an aircraft could be engaged; however, this was substantially simplified for the purpose of our experiment and required participants only to select the aircraft they wanted to neutralize and then click on the “Engage” button to launch the missile. The weapon was represented by a small white dot on the radar screen. Only one anti-aircraft missile projectile could be airborne at one time.

Finally, participants needed to be sensitive to changes occurring in the operational space in addition to performing the other three subtasks. Each aircraft, when appearing on the radar screen, had a status that could be either non-hostile or uncertain. Over the course of a scenario, some aircraft could change their status from non-hostile or uncertain to become hostile if they displayed 4–5 threatening cues. Such changes were considered critical and required detection in order to avoid the hostile aircraft attacking the frigate. All critical changes were accompanied by a visual transient on the radar screen that is a change in the aircraft speed and/or direction. Selection could be made using the radar, or CHEX if available. If no actions were made on a hostile aircraft within 15 s (the minimal time delay between two critical changes), the change was considered undetected. Since the occurrence of a ship hit would not necessarily indicate that a critical change had been missed (other factors may contribute to ship hits), this was not used as a criterion for change detection. There were also non-critical changes that could be either: (1) a non-hostile or an uncertain aircraft changing speed and/or direction; (2) a non-hostile aircraft becoming uncertain; and (3) a non-hostile aircraft becoming an uncertain and changing speed and/or direction.

#### CHEX tool

In the CHEX condition, the tool was positioned to the right of the radar screen (Fig. 2, Area 4). As in Smallman and St. John (2003), the role of CHEX is to automatically detect and permanently store every change that occurs in the airspace. The apparition of change-related information in the table was not accompanied by an auditory alert. The table is separated into three sortable columns: time, ID, and changing parameter(s). Each time a change occurred (critical or not), CHEX logged all the modified parameters, one below the other, with the corresponding time, aircraft ID, and description. The next change was logged at the top of the table and previous changes moved towards the bottom of the table. Given the large number of changes accumulated in the table, CHEX had a scroll bar to allow participants to consult changes that had occurred at the beginning of a scenario and were therefore listed at the bottom of the table. The CHEX automation is fixed as its functionalities cannot be turned off by the operator and its table cannot be hidden or minimized on the display. As all changes are logged (critical and otherwise) and there is no further filtering of this information, the role of CHEX is to aid information acquisition rather than to provide any deeper analysis of the change. Participants can refer to CHEX at any time to determine whether a particular aircraft has undergone a certain type of change and when this change happened. A dynamic visual linking interconnects CHEX and the radar screen enabling the selection of an aircraft on either display, which highlights information relative to the selected aircraft in both displays.

#### Eye tracking

Eye movements were recorded with a Tobii T1750 eye tracker at a sampling rate of 50 Hz. Participants were seated in front of a computer monitor at a distance of 60 cm. Infrared eye tracking cameras are integrated into this monitor allowing participants to freely move their head. Each fixation had to last at least 100 ms to be recorded by the eye tracker and the fixation field corresponds to a 50-pixel radius circle. The functional field of view of the participant thus corresponds to a visual angle of approximately 2°. As well as analyzing fixations in the AOIs illustrated in Figs. 1 and 2, we also assessed fixations on specific aircraft during the 15 s following a change; that is, a fixation on a critically changed aircraft during that time window would be classified as a fixated rather than a non-fixated change. A critical change was considered fixated if the aircraft which underwent the change (or the associated change entry in the tool table when available) fell within 50 pixels of the center point of a fixation at least once during the 15-s post-change period and if this fixation lasted at least 100 ms.

#### Procedure

At the beginning of the experiment, participants were given a short tutorial to familiarize themselves with the context of the simulation and their tasks. This tutorial also informed participants assigned to the CHEX condition about the utility of the tool. After the instructions, they were required to perform the threat evaluation and immediacy judgments from nine static screenshots to confirm their understanding of the tasks. They then familiarized themselves with the microworld’s dynamic environment through two training sessions which comprised four 3-min scenarios. After calibrating the eye-tracking system, participants performed four randomly presented experimental blocks separated by 5-min rest periods. Each block comprised four 4-min scenarios of similar difficulty, presented randomly, for a total of 64 min of experimental testing. Each test scenario included a set of 27 aircraft (11 non-hostile, 8 uncertain, and 8 hostile) varying in speed and trajectory on the radar. A total of 33 changes occurred during a test scenario: 25 were non-critical and eight were critical. A maximum of 10 aircraft could appear on the radar screen at any one time. Overall, a participant had to detect 128 critical changes. After each scenario, participants answered two questions aloud regarding the mental and temporal demand indices from the NASA-TLX subjective workload questionnaire: (1) “How much mental and perceptual activity was required (e.g., thinking, deciding, calculating, looking, searching, etc.)? Was the task easy or demanding, simple or complex?” and (2) “How much time pressure did you feel due to the rate or pace at which the task occurred?” Participants indicated their perceived level of mental load and time pressure on a Likert-type scale of 1–10.

### Results and discussion

CHEX and control groups were compared according to three aspects of operator activities: (1) change-detection performance; (2) subjective mental workload; (3) usage of CHEX; and (4) TEWA performance. The alpha level was set at .05.

#### Overall change detection

**Fig. 3:**
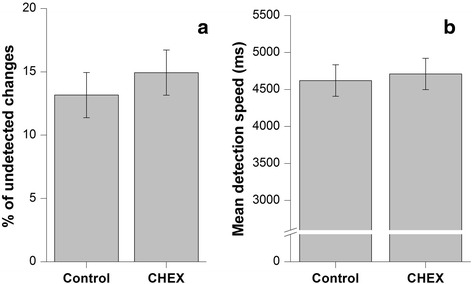
Mean percentage of undetected changes (panel **a**) and mean detection speed (in ms; panel **b**) for the control and CHEX conditions. *Error bars* represent 95% within-subject confident intervals calculated with Masson and Loftus’s (2003) method

Overall, 14.9% of critical changes were missed in the CHEX group and 13.17% in the control group (Fig. 3a), thus confirming the finding that complex, dynamic situations are vulnerable to CB (DiVita et al., 2004; Durlach & Chen, 2003; Smallman & St. John, 2003; Vachon et al., 2012). There was no significant difference in the number of undetected changes between the two conditions, *t*(49) = −1.040, *P* = 0.303, *d* = −0.29 (Fig. 3a), and similarly, the speed of detection did not differ significantly between groups, *t*(49) < 1 (Fig. 3b). These results indicate that—contrary to the findings of St. John et al. (2005)—CHEX failed to improve the detection of critical changes in the airspace.

#### Fixated versus non-fixated undetected changes

**Fig. 4:**
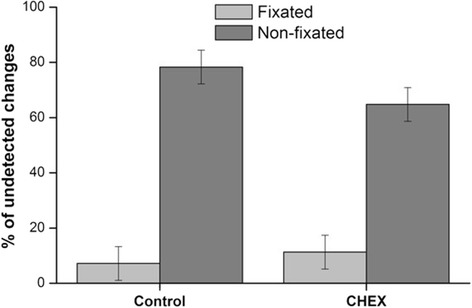
Mean percentage of undetected changes according to whether they were fixated or not for the control and CHEX conditions of Experiment 1. *Error bars* represent 95% within-subject confident intervals calculated with Masson and Loftus’s (2003) method

The undetected changes were further examined according to whether or not they had been fixated in the 15-s post-change interval. Overall, the mean percentage of critical changes that were fixated was 89.1% for the control condition and 88.3% for the CHEX condition. In Fig. 4, we compared the rate of undetected changes that were fixated with the changes that were missed and non-fixated, for each condition, using a 2 (fixation: fixated versus non-fixated) × 2 (condition: control versus CHEX) mixed ANOVA. This analysis revealed a significant main effect of fixation, *F*(1, 49) = 210.571, *p* < 0.001, η^2^_p_ = 0.811, with a greater percentage of undetected changes that were not fixated than were fixated (Fig. 4). There was no main effect of condition, *F*(1, 49) = 1.207, *p* = 0.277, η^2^_p_ = 0.024, but more importantly, the interaction effect was significant *F*(1, 49) = 4.212, *p* = 0.045, η^2^_p_ = 0.079. Regarding non-fixated changes, simple effect tests showed that there was no significant difference between the two conditions in terms of the percentage of changes undetected (*p* = 0.120). However, the CHEX condition produced a higher percentage of undetected changes that were fixated than the control condition (*p* = 0.003), thus CHEX increased the likelihood that a change—although attended—could remain undetected.

Given that the addition of the CHEX table produced a change in luminosity and contrast to the participants’ interface when compared to the interface of the control condition—a difference that could affect pupil dilation—we used dwell time on the changing aircraft for assessing attentional processing during fixations instead of measuring the pupil diameter (Poole & Ball, 2006). Critical changes that had been fixated were then examined according to whether or not they were detected, to verify the impact of CHEX on dwell time on the changed aircraft (using the radar and/or the tool) during the 15-s post-change interval (see Table 1). A 2 (detection: detected versus undetected) × 2 (condition: control versus CHEX) mixed ANOVA revealed a significant effect of detection *F*(1, 49) = 226.612, *p* < 0.001, η^2^_p_ = 0.822, with greater dwell time on the radar screen and/or the CHEX for detected changes compared to undetected changes. The main effect of condition was also significant, *F*(1, 49) = 8.637, *p* = 0.005, η^2^_p_ = 0.15, showing that participants in the CHEX condition spent more time looking at the changing aircraft on the radar and/or on the tool during the 15-s post-change interval than participants assigned to the control condition. Moreover, the ANOVA revealed a significant interaction effect, *F*(1, 49) = 4.018, *p* = 0.05, η^2^_p_ = 0.076, with simple effect tests showing a significant difference between the CHEX and control condition regarding the dwell time on changing aircraft for undetected changes (*p* < 0.001), but not for detected changes (*p* = 0.166). This indicates that both the presence and the usage of CHEX is indeed responsible for the higher number of attended changes that remained undetected and therefore the exacerbation of the attention-failure source of CB (cf. Vachon et al., 2012). During this complex and demanding task, the further addition of the CHEX tool to the interface may have exceeded participants’ attentional capacity, causing a temporary breakdown in attentional processes and increasing the likelihood that a critical change would be missed.

**Table 1:** Dwell time (mean + SD) on changing aircraft using the radar and/or the CHEX during 15-s post-change interval in Experiment 1

	Control condition	CHEX condition
Using the radar (in ms)		
Undetected changes	666.57 (264.07)	573.87 (253.6)
Detected changes	1667.93 (439.76)	1514.48 (465.33)
Using the tool (in ms)		
Undetected changes	N/A	524.17 (414.2)
Detected changes	N/A	349.32 (369.82)
Total		
Undetected changes	666.57 (264.07)	1098.03 (439.76)
Detected changes	1667.93 (439.76)	1863.84 (551.51)

#### Subjective workload

Measures of subjective workload were taken using the two subscales of the NASA-TLX. An independent-samples *t* test revealed that the CHEX condition was associated with a higher level of perceived mental load (M: 7.17, SD: 1.29) when compared to that obtained in the control condition (M: 6.29, SD: 1.30), *t*(49) = −2.405, *p* = 0.020, *d* = −0.697. However, there was no significant difference between the average level of time pressure reported by participants assigned to the CHEX condition (M: 6.83, SD: 1.69) and that perceived by participants in the control condition (M: 6.43, SD: 1.31), *t*(49) < 1. The CHEX had a detrimental effect on subjective workload as participants judged that their tasks were more demanding and complex and required more mental and perceptual activities in the presence of the tool than in its absence.

This suggests that participants placed in a multitasking dynamic situation may not have been able to make full sense of the change-related information contained in the CHEX table due to limited cognitive resources, making it less effective than in studies using a single-task environment. If, as suggested by Vachon et al. (2012), the extraction of visual information can be momentarily degraded by the high cognitive demands of the situation, it is likely that the extraction of information logged in CHEX suffers the same complications.

#### CHEX usage

Although the support tool failed to reduce the incidence of CB, it is interesting to gauge the extent to which participants may have thought that CHEX was helpful to the task by assessing their usage. Table 2 shows that usage of the DSS was sporadic; participants fixated on the CHEX tool for just 2.42% of scenarios. Such an additional information load in an already demanding context may have prevented sufficient time or attentional resources to attend properly to the supplementary tool, explaining why CHEX was used only sporadically.

**Table 2:** Behavioral and ocular metrics (mean + SD) of the CHEX usability for Experiment 1 and 2

	TEWA + CHEX	Change detection only + CHEX
Mean number of aircraft selections		
On the tool	96.76 (91.23)	724 (765.813)
On the radar	679.8 (191.49)	1119.95 (1062.7)
Selections on the tool (%)	12.5 (11.55)	45.54 (45.63)
Eye movements on the tool		
Overall dwell time (%)	2.42 (2.45)	11.5 (10.95)
Fixations (%)	3.92 (3.49)	28 (22.33)

Because data were averaged across scenarios, it is possible that the “overall” sporadic use of CHEX may be due to participants abandoning the tool during the course of the experiment. To test this possibility, we contrasted the three “usage” metrics across the four blocks of test scenarios using repeated-measures ANOVAs. A significant difference was observed across test blocks in terms of the percentage of selections made using CHEX, *F*(3, 72) = 2.897, *p* = 0.041, η^2^_p_ = 0.108, as selections seemed to increase between test 1 (M: 12.7%, SD: 12.27%) and test 2 (M: 14.2%, SD: 12.71%) and seemed to decrease and to remain the same in the last two tests (M: 11.93, SD: 1123%; M: 11.26%, SD: 11.75%, respectively). However, none of the multiple comparison tests performed were significant. The results were more clear cut for the other CHEX usage metrics: the percentage of fixations on the CHEX table did not change over time, *F*(3, 72) = 1.47, *p* = 0.230, η^2^_p_ = 0.058 (overall mean: 3.98%), nor the dwell time on the tool, *F*(3, 72) < 1 (overall mean: 23.19 s). These results indicate that CHEX was used (sporadically) throughout the entire experiment.

#### TEWA performance

**Fig. 5:**
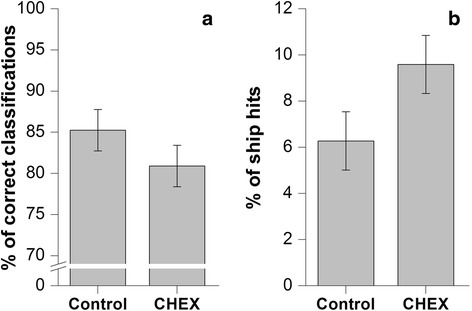
Mean percentage of correct classifications (panel **a**) and ship hits (panel **b**) for the control and CHEX conditions of Experiment 1. *Errors bars* represent 95% within-subject confident intervals calculated with Masson and Loftus’s (2003) method

In line with our holistic assessment of CHEX, we assessed performance on the TEWA subtasks, namely threat evaluation (percentage of correct classifications) and defensive effectiveness (percentage ship hits by a hostile aircraft). According to an independent-samples *t* test, there was no significant difference between the average level of classification accuracy obtained in the CHEX condition and that observed in the control condition, *t*(49) = 1.736, *p* = 0.089, *d* = 0.48 (Fig. 5a). However, the presence of CHEX significantly increased the percentage of ship hits relative to the control condition *t*(49) = −2.783, *p* = 0.008, *d* = −0.77 (see Fig. 5b). The addition of CHEX to the original interface impaired TEWA performance meaning that the own ship was more vulnerable to hostile threats with this support tool than without.

The increase in subjective workload in the CHEX condition would seem to provide some explanation for the associated decrease in TEWA performance in the presence of the tool. Such a result suggests that the presence of the DSS on the display provided an additional source of information to monitor that could have increased the apparent cognitive load of the task (Perry, Wiggins, Childs, & Fogarty, 2013). Indeed, introducing a table listing all the changes (critical and non-critical) occurring in the complex dynamic situation increased the amount of visual information to process before making a decision. It has also been shown that the mere presence of a tool or modifying a part of an interface can change the way a task is performed when compared to that observed beforehand (McCrickard, Catrambone, Chewar, & Stasko, 2003; Rousseau, Tremblay, Lafond, Vachon, & Breton, 2007). Therefore, this extra burden and pressure that the addition of CHEX put on participants may have affected their efficacy in making precise and timely decisions with regard to weapon assignment, thus leaving the own ship more exposed to attack (ship hits).

## Experiment 2

The finding that CHEX did not improve change detection in our task would seem to be at odds with the work of St. John et al. (2005) which demonstrated a clear reduction in CB with CHEX. Although both our study and that of St. John, Smallman, and Manes used dynamic, complex, and fairly realistic air-warfare microworlds, we suggest that the discrepancy may lie with the general task demands placed upon participants in each context; that is, whether detecting changes was the only task to perform or whether it formed part of a more complex, higher-order task. In Experiment 2, to test this hypothesis, we modified the S-CCS interface to remove elements associated with the TEWA task and participants’ only requirement was to identify critical changes that were associated with a classification change in the threat level of an aircraft. If CHEX improved change detection in this modified S-CCS context, thus replicating the results of St. John, Smallman, and Manes, it would suggest that the ability of CHEX to reduce CB is dependent upon the wider task requirements. The CHEX external aid was the same in Experiment 2 as that in Experiment 1, and participants used the information provided by the tool in the same manner in both experimental settings, for detecting and identifying critical changes.

### Method

#### Participants

Thirty-nine students from Université Laval took part: 20 were assigned to the control condition (10 men; mean age, 24.5 years) and the others were assigned to the CHEX condition (9 men; mean age, 26.3 years). None had taken part in Experiment 1.

#### Task

**Fig. 6:**
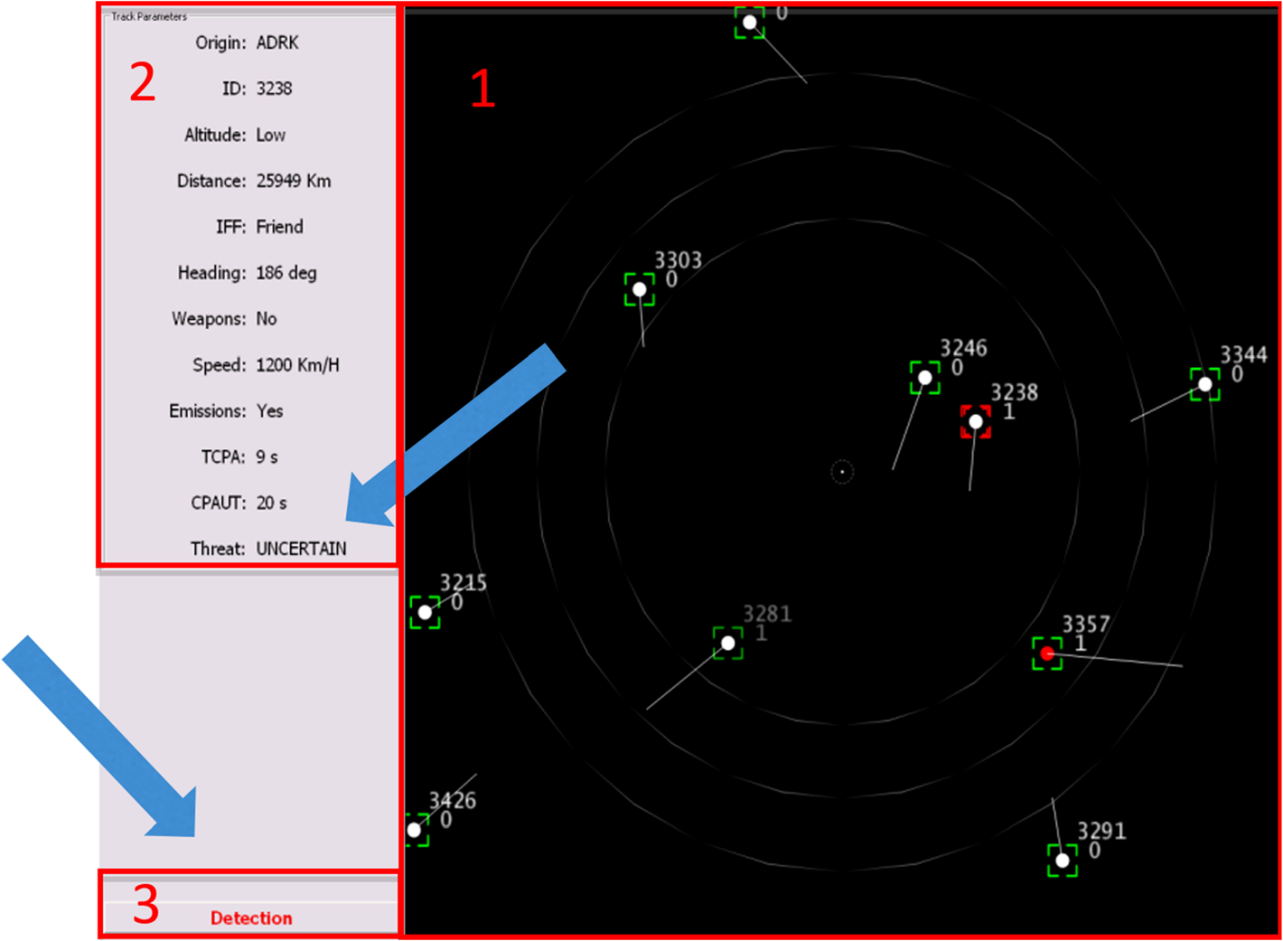
S-CCS display in the control condition of Experiment 2. *Arrows* point to the changes made to the parameter list and action buttons in order to adapt the interface to the context in which change detection was the only task to perform

Experiment 2 used the same microworld as previously, but with the difference that participants performed only one task: a change-detection task whereby they had to press a “Detection” button (see Fig. 6) as soon as they detected a critical change, that is, when an aircraft changed from a non-threatening (i.e. non-hostile or uncertain) to a threatening (i.e. hostile) status. Although participants had to select an aircraft and refer to the “threat” parameter of the list (see Fig. 6) to verify its status, each critical change was accompanied by a change in the speed and/or direction of that aircraft visible on the radar screen, as in the previous experiment. When pressing the “Detection” button, the white dot representing the aircraft then turned red and corresponding lines in CHEX, when available, turned gray. This was done to help participants to keep track of which critical changes they had already detected and reported. There were again eight critical changes per scenario.

In order to discourage participants from systematically clicking on the “Detection” button as soon as they saw a “hostile” status in the parameter list and thus to focus only on changing—newly hostile—aircraft, two hostile aircraft acting as false alarms were present on the radar screen at the beginning of each experimental scenario. The dot representing these hostile aircraft was white as for all other aircraft appearing on the radar. Participants were aware of these “non-changing” hostile aircraft and therefore knew that the presence of a hostile aircraft did not automatically imply a critical change to the situation. Participants were asked not to detect and report these non-changing hostiles and to focus exclusively on new threats. When participants noticed a change in direction and/or speed, they had to select the associated aircraft and verify the threat parameter to determine whether it constituted a critical change (i.e. a change from a non-threatening to a hostile status) or not (i.e. the aircraft was not hostile or had been hostile since the beginning of the scenario).

#### Microworld

**Fig. 7:**
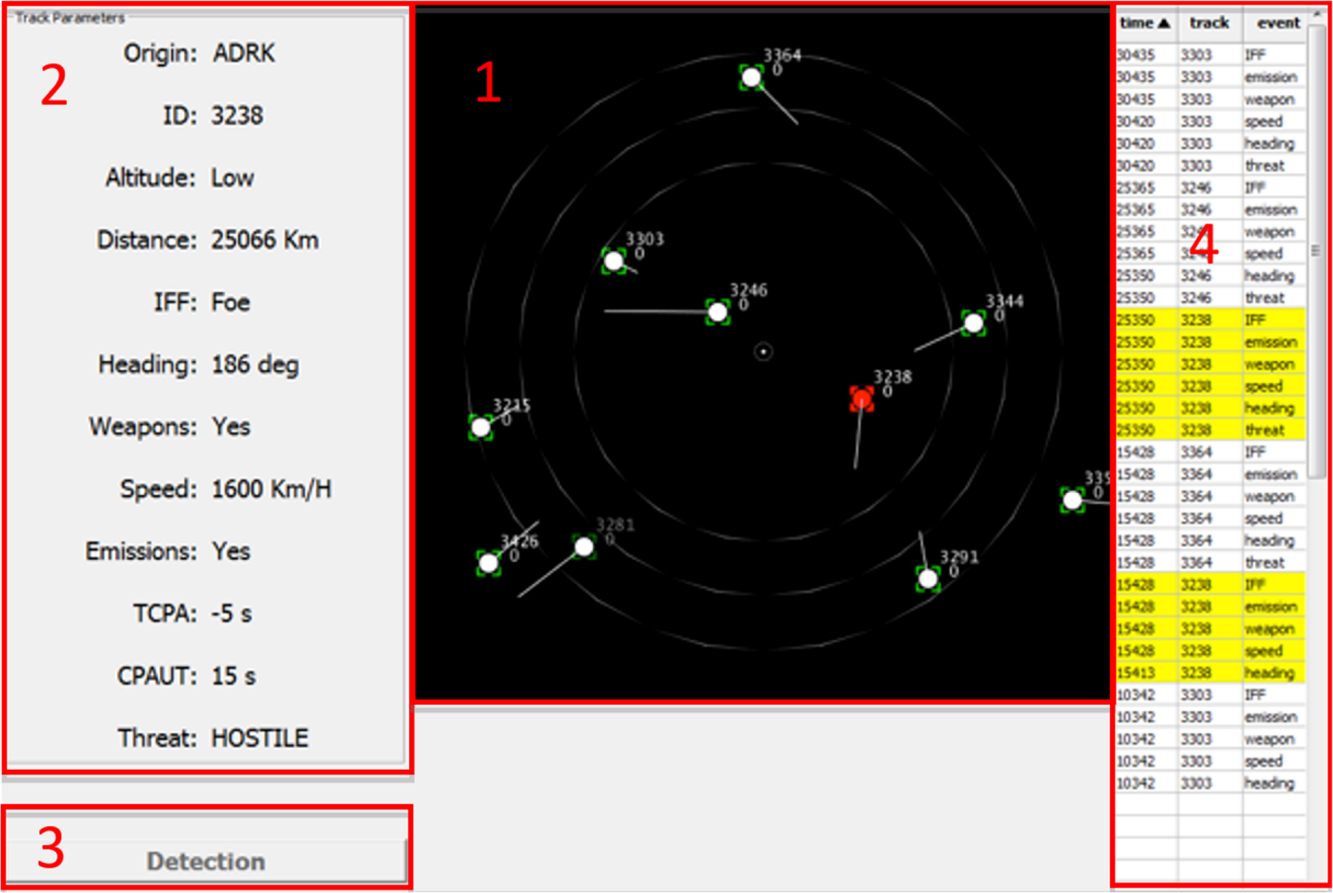
S-CCS display in the CHEX condition of Experiment 2

The microworld was identical to that used in previous experiments, except for the following features. As shown in Fig. 6, the attribute “threat,” which designates the actual threat level of the selected aircraft, was added to the parameter list. This was because participants still had to detect changes in aircraft status—i.e. notice new hostile aircraft—but were no longer required to classify the aircraft appearing on the radar. Aircraft classification was thus performed by the computer and the result of this classification was provided to participants through the “threat” parameter. Therefore, participants had to detect status changes based on visual cues (i.e. change in speed/direction) and/or CHEX (when available) and determine whether these changes were critical or not using the “threat” parameter. The remaining parameters were now irrelevant to the task. The “Detection” button replaced the “Engage” button which, in previous experiments, was used to launch anti-aircraft missiles. This button allowed participants to explicitly report the critical changes. The immediacy and classification buttons were also removed from the interface as change detection was now the sole task to perform. In the control condition (see Fig. 6), participants performed change detection with no additional support whereas in the CHEX condition (see Fig. 7), participants had access to CHEX.

### Results and discussion

#### Overall change detection

**Fig. 8:**
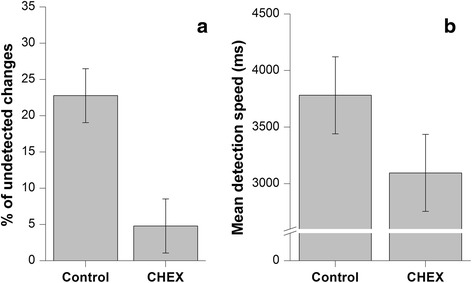
Mean percentage of undetected changes (panel **a**) and mean detection speed (in ms; panel **b**) in each condition of Experiment 2. *Error bars* represent 95% within-subject confidence intervals calculated with Masson and Loftus’s (2003) method

As shown in Fig. 8a, the percentage of undetected changes^1^ was significantly higher in the control condition than when CHEX was available, *t*(37) = 4.945, *p* < 0.001, *d* = 1.596. Participants were also significantly faster to detect critical changes in the CHEX condition than in the control condition, *t*(37) = 2.059, *p* = 0.047, *d* = 0.66 (see Fig. 8b). In line with previous findings by St. John et al. (2005), CHEX can greatly improve change detection when this is the only task to perform. In such a context, CHEX becomes a powerful tool to reduce CB.

#### Fixated versus non-fixated undetected changes

**Fig. 9:**
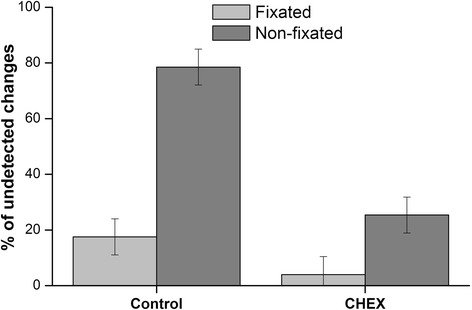
Results from Experiment 2: mean percentage of undetected changes according to whether they were fixated or not for the control and CHEX conditions. *Error bars* represent 95% within-subject confident intervals calculated with Masson and Loftus’s (2003) method

Overall, the mean percentage of critical changes that were fixated was 90.6% for the control condition and 89.9% for the CHEX condition. As for Experiment 1, the rate of detection failures for fixated and non-fixated changes was computed separately and then analyzed using a split plot 2 × 2 ANOVA (fixation × conditions). Three participants in the CHEX condition were removed from the analysis as they fixated all the critical changes and so would have been considered as missing data. The ANOVA revealed significant main effects of fixation, *F*(1, 34) = 84.219, *p* < 0.001, η^2^_p_ = 0.712, and of condition, *F*(1, 34) = 35.345, *p* < 0.001, η^2^_p_ = 0.510 (see Fig. 9). More importantly, we obtained a significant two-way interaction, *F*(1, 34) = 19.447, *p* < 0.001, η^2^_p_ = 0.364. This interaction arose because, while there were fewer undetected changes that were fixated than not, this difference was significantly attenuated in the presence of CHEX relative to the control condition (*p* < 0.001). When change detection was the only task to perform, CHEX was successful in reducing both sources of CB, particularly those changes that were missed because they were never attended.

Critical changes that had been fixated were then examined according to whether or not they were detected, to verify the impact of CHEX on dwell time on the changed aircraft (using the radar and/or the tool) during the 15-s post-change interval (see Table 3). Five participants were removed from the analysis as they detected all the critical changes that they fixated and so would have been considered as missing data. A 2 (detection: detected versus undetected) × 2 (condition: control versus CHEX) mixed ANOVA indicated that there was no main effect of detection, *F*(1, 32) = 1.781, *p* = 0.191, η^2^_p_ = 0.053, nor interaction effect, *F*(1, 32) < 1. However, the analysis revealed a significant effect of condition, *F*(1, 32) = 9.970, *p* = 0.003, η^2^_p_ = 0.238. Participants assigned to the CHEX condition spent more time looking at the changing aircraft on the radar screen and/or on the tool during the 15-s post-change interval than participants in the control condition. As shown in Table 3, in the CHEX condition, participants spent the vast majority of the 15-s post-change intervals fixating the CHEX tool rather the radar display. In the change-detection-only context, it is clear that adding the CHEX log to the interface allowed the participants to spend more time analyzing the changing objects, which helped them to successfully detect the great majority of critical changes.

**Table 3:** Dwell time (mean + SD) on changing aircraft using the radar and/or the CHEX during 15-s post-change interval in Experiment 2

	Control condition	CHEX condition
Using the radar (in ms)		
Undetected changes	1154.32 (525.16)	275.74 (368.55)
Detected changes	1618.65 (457.36)	953.71 (416.81)
Using the tool (in ms)		
Undetected changes	N/A	2683.89 (2863.59)
Detected changes	N/A	2101.43 (2071.62)
Total		
Undetected changes	1154.32 (525.16)	2959.63 (2794.41)
Detected changes	1618.65 (457.36)	3055.14 (1987.58)

#### Subjective workload

With CHEX support, participants reported significantly lower levels of mental load (M: 6.02, SD: 2.13), *t*(36) = 2.038, *p* = 0.049, *d* = 0.661, and time pressure (M: 5.07, SD: 1.75), *t*(36) = 2.079, *p* = 0.045, *d* = 0.675, than without such support (M: 7.21, SD 1.41 and M: 6.20, SD: 1.59, respectively). It thus appears that, contrary to Experiment 1, CHEX can help to reduce subjective workload but only when this is the only task to perform. In such cases, the operator has available attentional capacity to be able to use the tool to their advantage; with the knowledge that changes are automatically logged in a table, perceptions of pressure and mental load are decreased because participants know they can always refer to the permanent record of CHEX to supplement what they see on the radar.

#### CHEX usage

As shown in Table 2, when change detection was the sole task, participants made significantly more aircraft selections using CHEX than when change detection was embedded within the TEWA task (Experiment 1), *t*(42) = −3.493, *p* = 0.001, *d* = −0.994. In Experiment 2, participants spent 11.5% of the experiment duration fixating CHEX, which is significantly higher than that in the CHEX condition of Experiment 1 (2.42%), *t*(42) = −3.563, *p* = 0.002, *d* = −1.149.

We performed repeated-measures ANOVAs on all “usage” metrics and found the percentage of selections using CHEX varied over time, *F*(3, 54) = 5.486, *p* = 0.022, η_p_^2^ = 0.234. The average percentage of selections using the CHEX was 41.59% (SD: 42.38%) for test 1, 46.62% (SD: 46.71%) for test 2, 47.29% (47.31%) for test 3, and 47.84% (SD: 47.88%) for test 4. Even though none of the multiple comparisons tests performed were significant, a trend analysis shows that the percentage of selections using CHEX increased linearly across the four test scenarios, *F*(1, 18) = 6.286, *p* = 0.022, η_p_^2^ = 0.259, suggesting that participants began to see the benefit in using the tool the more they became familiar with it. Dwell time on CHEX, *F*(3, 54) = 1.477, *p* = 0.231, η_p_^2^ = 0.076 (overall mean: 117.71 s), and percentage of fixations on the tool (*F* < 1; (overall mean: 27.35%) remained the same over time.

## General discussion

The objective of the present study was to test whether the efficacy of CHEX in supporting situation awareness maintenance could generalize to a context in which situational change detection served the higher goals of a primary task that comprised three other subtasks. However, far from supporting participants, results from Experiment 1 showed that CHEX increased the attentional source of CB, reduced defensive effectiveness, and led to higher levels of perceived mental workload. The success of a CHEX-like tool in augmenting change detection thus seems to depend very much upon the task in which it is used. Indeed, in line with the findings of St. John et al. (2005), CHEX was able to reduce CB within our naval air-warfare simulation when the detection of critical changes was the only task to perform (Experiment 2). In these circumstances CHEX can provide a useful log of changes to augment cognition and facilitate cross-referencing with the radar display.

We acknowledge that, with the introduction of a change-detection tool to the interface, the radar size varied across the two conditions of both experiments and may have impacted performance. However, even though in the CHEX condition the spacing between radar objects was slightly smaller than that observed in the control condition, the aircraft icon size remained the same and critical changes were always accompanied by the same visible change of speed and/or direction on the radar. These radar changes were obvious and unequivocal and aircraft were never occluded by another object. Therefore, we are confident that any impact of variations in radar size would have been negligible.

One recent model of attention behavior, NSEEV (noticing – salience, effort, expectancy, value; Steelman, McCarley, & Wickens, 2011; Wickens et al., 2009), can provide a useful theoretical basis for the current work on change detection. This model predicts scanning and noticing behaviors in a dynamic visual workspace by combining bottom-up factors of attentional control (e.g. the salience of a target) and top-down factors (e.g. the expectancy of an event occurring in a particular location, and the value or criticality of that information) and the effort needed to shift attention towards that item (eccentricity from current point of gaze). More recent work on the NSEEV emphasized on the interactions between salience, expectancy, and eccentricity in alert detection. Eccentricity includes factors pertaining to the target location within the visual scene, such as the acuity loss in the peripheral vision and the effort needed to direct attention towards the different regions of the scene, to explain increasing reaction times and error rates as a function of the distance between the target location and the gaze location of the participant. Steelman, McCarley, and Wickens (2013) showed that visual alerts appearing in low-probability locations not only were associated with poorer detection performance, but exacerbated the negative impacts of low-salience and eccentric targets. We consider how features of this model might explain noticing behavior in the current task and thus the extent to which CHEX hindered change detection rates under multitasking conditions.

### CHEX and sources of CB

The pre-attentive source of CB occurs when the participant never looks at the change (failure to direct attention towards a change; e.g. Henderson & Hollingworth, 2003; O’Regan et al., 2000; Rensink et al., 1997; Simons & Ambinder, 2005). On a large display featuring numerous aircraft on the radar, as well as a list of associated parameters, shifting attention between all areas of the screen can be effortful and items or events that are further away from the point of gaze are more likely to be missed (one factor of the eccentricity parameter of NSEEV; Steelman et al., 2011, 2013). Despite having an additional area of interest on the display in the case of the CHEX condition—which could have increased the chance that participants were looking in the wrong place at the wrong time—fixations on the tool were minimal and there appeared to be no difference between conditions in terms of changes missed because attention was directed elsewhere.

Directed attention is required for change detection, but it is not always sufficient (see Simons & Rensink, 2005; Beck et al., 2007); changes may also be missed due to a failure of attentional processes (Privitera, Renninger, Carney, Klein, & Aguilar, 2010; Vachon et al., 2012). According to this second source of CB, missing changes can result from a failure in selection the objects to encode in memory, making sense of the changes, or in making the appropriate action after the changes. Registering a change requires a mental comparison with that object’s previous state and in the current task, recollection and comparison of subtle changes in a dynamic rather than a static attribute may be particularly demanding (e.g. a change in speed of an already moving object). In the NSEEV model, the salience of the target is influenced by both static features (contrast of the item of interest and the environment; see Itti & Koch, 2000) and dynamic or motion features (moment-to-moment changes of static salience; see Loy, Xiang, & Gong, 2012; Yantis & Jonides, 1990). Indeed, the current task may be particularly vulnerable to CB in general, as there are no salient static or dynamic features that distinguish a critically changed aircraft from any other on the radar screen in any of the two conditions. This concurs with the higher dwell time on the changing objects during the post-change interval for unnoticed changes in the CHEX condition than in the control condition of Experiment 1. Participants devoted a greater amount of attentional resources in the attempt to extract, compare, and comprehend the change-related contextual cues displayed by the radar and the tool, but failed to consciously detect the attended changes.

The NSEEV model also suggests that the size of the functional field of view narrows under conditions of high workload, meaning that the current task—likely to require a broad attentional breadth in order to register the necessary dynamic changes, as well as perform the TEWA task consecutively—may be particularly vulnerable to increases in workload. The CHEX condition introduces a higher visual load, resulting in impaired detection of these dynamic changes. The CHEX table keeps a permanent list of every change (critical and non-critical) that has occurred as well as numerous attributes associated with it. This serves to increase the amount of information on the screen, but fails to improve the salience of the most important information as critical and non-critical changes are not distinguished from each other. Although use of the tool was sporadic, occasional monitoring of the changes list may have created a more demanding divided attention condition whereby participants intermittently shifted attention towards the cluttered table to check if important information might be gleaned from it (Rosenholtz, Li, Mansfield, & Jin, 2006). Moreover, the mere presence of CHEX on the screen may have increased workload through an overhead cognitive cost in deciding when—or indeed whether—to shift attention to the automated tool. This high workload combination (CHEX plus TEWA task) means that attentional focus is narrowed and reduces the probability that sufficient attention can be directed at a change in order to detect it (Perry et al., 2013).

In Experiment 2, the replica of the original CHEX then proved a useful support tool in reducing both types of CB. In a complex dynamic task situation, attention can often be directed towards other areas of the interface leading critical changes to sometimes go unnoticed as the more eccentric the changes occur from the participant’s point of gaze, the more the peripheral vision is degraded. CHEX permanent repository of changes can help to compensate for those momentary lapses of attention. When operator capacity allows, participants can then rely on CHEX to re-deploy attention to the information they need in order to report an initially undetected change. Importantly in this single-task rather than multitask environment, CHEX was also able to reduce the attentional source of CB relative to No-DSS: since the tool allowed for offloading—rather than detrimentally overloading—cognitive processes needed for extracting, comparing, and understanding the evolution of the situation, participants were better able to answer the calls for attention demanded by the critical changes. The high-load context of Experiment 1 likely narrowed attentional breadth (narrowing of functional field of view) making the detection of fixated dynamic changes more difficult thus compromising the ability to register, extract, and comprehend dynamic change information. In Experiment 2 however, CHEX actually reduced workload and so a broader attentional breadth may have better allowed for the processing of change-related information when a critically changed aircraft was fixated. Future work should apply the computational capacity of the NSEEV model to predict the detection rates in both with and without CHEX conditions of this study in order to confirm the influence of the bottom-up and top-down factors in the inefficacy of CHEX.

### TEWA task performance

The mere presence of CHEX on the S-CCS interface was enough to disrupt TEWA performance. It is possible that while cognitive resources were devoted to carrying out unnecessary actions such as crosschecking the veracity of a change notified by CHEX, an insufficient amount of resources were allocated to the other TEWA subtasks which might have resulted in scheduling impairment (Dixon & Wickens, 2006). As shown by several studies, the simple modification of a part of an interface can change information seeking and eye movement behaviors, as well as decision-making processes, and therefore can modulate how a task is performed (McCrickard et al., 2003; Rousseau et al., 2007). In accordance with this finding, modifying the S-CCS interface by introducing CHEX could have been enough to change the way participants conducted TEWA, hence leading to poorer defensive effectiveness.

### Sporadic use of CHEX

Because usage of CHEX did not vary over time, its sporadic use cannot be attributed to a lack of training with the tool—whereby one would have expected usage to increase with familiarity across the 88 min experiment—or to participants’ mistrust in CHEX automation (Parasuraman & Riley, 1997), whereby usage might have decreased given the number of non-critical or ‘false alarm’ changes logged in the table creating a sense of unreliability (a cry-wolf effect; Brenitz 1983; Wickens et al., 2009b). More likely is that given the high cognitive workload imposed by the concurrent TEWA subtasks (Experiment 1) and the fact that making sense of the table required quite an investment in time and resources, participants lacked the necessary cognitive resources to devote to the tool consistently over the whole course of the experiment (Wickens, 2002). There may have been a feeling that by devoting too much time to the table, they would miss key information displayed on other critical areas of the interface (e.g. radar) that was necessary to perform the TEWA activities. On the other hand, in a single-task context (Experiment 2), usage of CHEX was greater as more cognitive resources were available for processing and understanding critical change-information from the CHEX table.

The NSEEV model parameters could also shed light on the disuse of the tools, in the way that participants do not want to invest the effort (eccentricity) in shifting attention to the right-hand side of the screen, when the information they are using to perform the TEWA task can be found centrally or on the left-hand side (radar screen and parameters list). The NSEEV model discourages large attentional shifts by including a spatial filter that reduces the salience of peripheral stimuli and therefore mimics the inhibitory properties of effort and poor acuity of long visual saccades. While the radar and parameters lists are essential for the focal task of TEWA, CHEX is perhaps seen as an optional addition that is too effortful to process under these high-load conditions and so participants rarely shift their attention towards it. Moreover, participants might have privileged the radar screen when searching for a critical change as they were expecting to notice it more easily in this part of the interface rather than in the CHEX table. The combination of the high eccentricity and the low expectancy associated with the CHEX tool might explain the sporadic use of the CHEX. Indeed, in Experiment 2 when change detection was the only task to perform, there was less emphasis on the parameters list on the left-hand side of the screen, so participants could devote more time to the tool on the right-hand side without requiring long effortful attentional reallocation between the two. The longer dwell times over the CHEX table show that participants expected to find critical changes using the tool.

NSEEV also emphasizes the importance of value (or task-relevancy and priority) of the information displayed in the different portions of an interface in determining the likelihood of change detection and participants may consider certain areas of the screen to be more useful for change detection than others (Horrey, Wickens, & Consalus, 2006). It is possible that the value of the CHEX table was lower when it was used within the multitasking context as it was relevant and directly useful to only one subtask. Participants might have prioritized the use of the radar and the parameters list as they were relevant for all their subtasks. Their propensity to “neglect” CHEX may have also been amplified by their subjective tendency to rely on tools that maintain the high-fidelity spatio-temporal realism of the visual scene (naïve realism; Smallman & St. John, 2005), preventing them from considering the true utility of CHEX to help perform their task. Indeed, the transposition of the events occurring in the situation into verbal entries displayed in the cluttered CHEX table may have been less appealing or less valuable for change detection than presenting them over a geospatial display. Participants may have believed that any critical changes would be more easily apparent and detected on the radar that displays speed/direction information dynamically, than by sorting through a list of written information. In Experiment 2, given that the focus was solely on change detection and not on the performance of other subtasks, participants may have placed a higher level of value on the change detection tool provided for them specifically to facilitate the task and thus usage was higher. Participants’ judgment of where the most useful information could be gleaned from would have influenced their gaze behavior.

### Solutions for preventing CB

The NSEEV model can guide us towards a number of ways to prevent CB. Increasing the salience of critical changes is a fundamental step (see Loy et al., 2012; Zhang, Yuan, Zheng, Sheng, & Liu, 2010): given that CHEX stores change-related information for all objects in a dynamic situation, the table can rapidly become cluttered (Rosenholtz et al., 2006). The design could perhaps benefit from the implementation of an algorithm that would prioritize key information (see St. John et al., 2005). Instead of the default chronological ordering, an algorithm could process visual objects in such a way that the most important changes would be displayed at the top and those less important would appear at the bottom of the table (see Yeh & Wickens, 2001) or the level of importance could be varied through color intensity (see St. John et al., 2005b) to further increase the static salience of critical changes. A prioritization algorithm could reduce the negative impact of this clutter by enabling more effective filtering of critical from non-critical information (see Carver & Turoff, 2007; Hegarty, 2011), decreasing the saliency of entries less likely to be critical (see Wickens, Ambinder, & Alexander, 2004) and thus increasing the value of the information held in the table. However, any form of automatic prioritization could also generate errors. Current technology is not able to achieve a perfect hit rate (e.g. all critical changes are detected and correctly ordered) and false positives could not be completely avoided (Thomas & Rantanen, 2006). Automated prioritization systems, optimal or not, are also prone to the well-known risks of automation such as operator mistrust and overreliance (see Parasuraman, Sheridan, & Wickens, 2008; Parasuraman & Riley, 1997).

NSEEV predicts that detection of an event is related to the eccentricity of the target location in the visual scene. One potential solution then would be to integrate the real-time change tracking functionality within the radar view rather than adding a separate tool on the display (see Loy et al., 2012; Zhang et al., 2010). This would reduce the need for shifting gaze across a wide area and for moving the focus of attention across very different display formats (see Athènes, Chatty, & Bustico, 2000). Given the inefficacy of an external aid in the current multitasking context, radar-integrated notification systems should be tested in future studies. Indeed, such systems may seem intuitively more appealing and valuable to operators as they believe they are more likely to notice changes when presented with a dynamic spatial display than a written list. Having this naïve realism would perhaps mean the operator assigning more value to the radar-integrated change-tracking tool than a list of changes and so events may be more easily detected. In air-traffic control, visual notifications to aircraft that require an immediate response from the controller can emerge from the radar display (e.g. Imbert et al., 2014). These notifications are visual alerts to single critical events whereas the CHEX tool stores and displays a list of changes to all objects that may require a response. Whether an alert-like notification or a change history table is best suited depends on the nature of the task, its tempo (frequency of non-critical and critical events), and also on the level of discriminability and reliability of the filtering algorithm-based system.

## Conclusion

The current study suggests that decision aids for use in multitasking contexts must be designed to fit within the available workload capacity of the user and the timeliness of the situation so that they may truly augment cognition. Here we provide a demonstration that sometimes a system designed to help a specific users’ cognitive function can also backfire and have unintended consequences such as decision-making errors (Trafton & Ratwani, 2014). By providing a comprehensive cognitive assessment of the support system, we clearly show that changing the nature and the demands of a dynamic decision-making task made a tool—proven effective to prevent CB within a change-detection-only task environment—ineffective once used in a multitasking context. The efficacy of CHEX did not generalize to an environment that replicates the multitasking conditions and the attentional demands of real C2 operations often observed in air traffic control, surface warfare and security surveillance. In relation to the CB phenomenon, it is clear that its rate of occurrence is likely to be very high in dynamic real-life monitoring tasks and that cognitive support to prevent this problem still remains to be developed and validated.

Moreover, the current study provides further empirical evidence of the existence of two sources of CB as previously shown by Vachon et al. (2012). Therefore, the evaluation of a change-detection support system could involve a test of its efficacy to prevent both sources of CB by employing eye tracking. An efficient change-detection tool must not only be able to help an operator detect changes, but also identify and understand them, while limiting the cost in attentional resources needed to process the information it provides, especially in such contexts that operators’ cognitive resources are already overburdened.

## Abbreviations

ANOVA: Analysis of variance
AOI: Area of interest
C2: Command and control
CB: Change blindness
CHEX: Change History Explicit
DSS: Decision support system
NASA-TLX: NASA task load index
N-SEEV: Noticing Salience Effort Expectancy Value
S-CCS: Simulated Combat Control System
TEWA: Threat Evaluation and Weapon Assignment

## Footnotes

1 Although participants in Experiment 2 had only to perform change detection, the percentage of undetected changes observed in the control condition was nevertheless much higher than in the control condition of Experiment 1. Such a counterintuitive finding may have arisen from an underestimation of the percentage of undetected changes in the previous experiment. Indeed, when change detection was embedded within the TEWA task, correct detections were indexed by the timely actions made on the changed aircraft. It is thus possible in such a context that some changes never perceived by the participants were nonetheless recorded as detected because participants selected within the 15-s post-change period the changed aircraft on the basis of the requirements of the other subtasks they had to perform (and not because they had truly noticed the change). Such an underestimation is impossible when change detection is the sole task to perform.
